# Proteomics and synaptome mapping to uncover diversity in excitatory synapses

**DOI:** 10.1093/jb/mvag056

**Published:** 2026-07-20

**Authors:** Takeshi Kaizuka

**Affiliations:** Genes to Cognition Programme, Institute for Neuroscience and Cardiovascular Research, University of Edinburgh, Chancellor’s Building, 49 Little France Crescent, Edinburgh BioQuarter, Edinburgh EH16 4SB, UK

**Keywords:** excitatory synapse, postsynaptic density, proteomics, synaptome mapping, neurological and psychiatric disorders

## Abstract

Synapses are the fundamental computational units of the brain. Although synapses are classically classified according to the neurotransmitter they use, glutamatergic excitatory synapses also exhibit marked heterogeneity in their molecular composition and structure. Understanding this diversity requires comprehensive approaches that go beyond small-scale, candidate-based analyses of synapses. Proteomic analyses of biochemically enriched synaptic fractions have provided unbiased, quantitative measurements of synaptic protein composition, revealing differences across brain regions, cell types, developmental stages, and physiological and pathological states. Proteomics has also revealed the composition and remodeling of synaptic protein complexes, as well as changes in post-translational modifications. In parallel, synaptome mapping, an imaging-based approach for the large-scale *in situ* analysis of synapses across the brain, has emerged as a form of spatial omics. In this approach, synapses labeled with a small set of synaptic proteins are systematically imaged across the brain. Synaptome mapping reveals whole-brain variation in synapse molecular identity and structural features, including differences in synapse size, shape, and spatial distribution *in vivo*. Together, these approaches show that excitatory synapses are diversified across anatomical, cellular, developmental, activity-dependent, and disease-related contexts. These complementary perspectives will advance our understanding of the synaptic mechanisms underlying neural computation, behavior, and brain disorders.

## Graphical Abstract

**Graphical Abstract:**
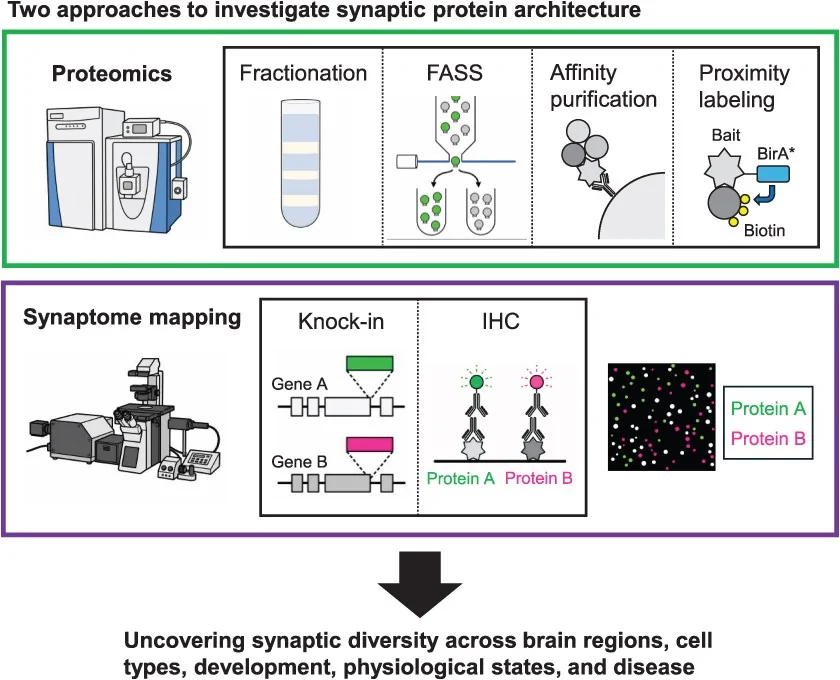

Synapses are junctions that mediate communication between neurons. Chemical synapses comprise pre- and postsynaptic specializations and can be broadly categorized as excitatory or inhibitory according to their effects on postsynaptic activity. Glutamatergic excitatory synapses are major computational units of the mammalian brain, converting presynaptic action potentials into postsynaptic electrical and biochemical signals (*1–4*). Dendritic spines are small, actin-rich protrusions that form the postsynaptic compartments of many glutamatergic excitatory synapses (*1–3*) (Fig. 1A). Excitatory neurotransmission is initiated by calcium-dependent vesicle fusion and glutamate release from the presynaptic terminal (*5*). Glutamate then binds to ionotropic receptors in the postsynaptic membrane, primarily α-amino-3-hydroxy-5-methyl-4-isoxazolepropionic acid (AMPA) receptors and N-methyl-D-aspartate (NMDA) receptors. AMPA receptors mediate the majority of fast excitatory currents, whereas NMDA receptors provide a coincidence-detection mechanism and allow calcium influx, linking synaptic activity to downstream biochemical cascades (*1**,*  *3*). The postsynaptic density (PSD) is a molecular assembly composed of scaffolding proteins, signaling enzymes, cytoskeletal regulators, and receptors (*1**,*  *2**,*  *6*) (Fig. 1B). Core scaffold proteins form a protein–protein interaction network that anchors glutamate receptors. The scaffold network also associates with intracellular signaling enzymes, providing a platform for signal transduction within the postsynaptic compartment. The resulting signaling processes contribute to synaptic plasticity, including structural remodeling mediated by reorganization of the actin cytoskeleton (*1–3*). The postsynaptic compartment provides a useful focus for examining the molecular complexity of excitatory synapses because it contains a large protein repertoire. A recent integration of mammalian synapse proteomic datasets identified 1354 proteins detected only in postsynaptic preparations, whereas 456 proteins were assigned to a presynaptic or shared pre−/postsynaptic category in the same analysis (*7*).

**Fig. 1:**
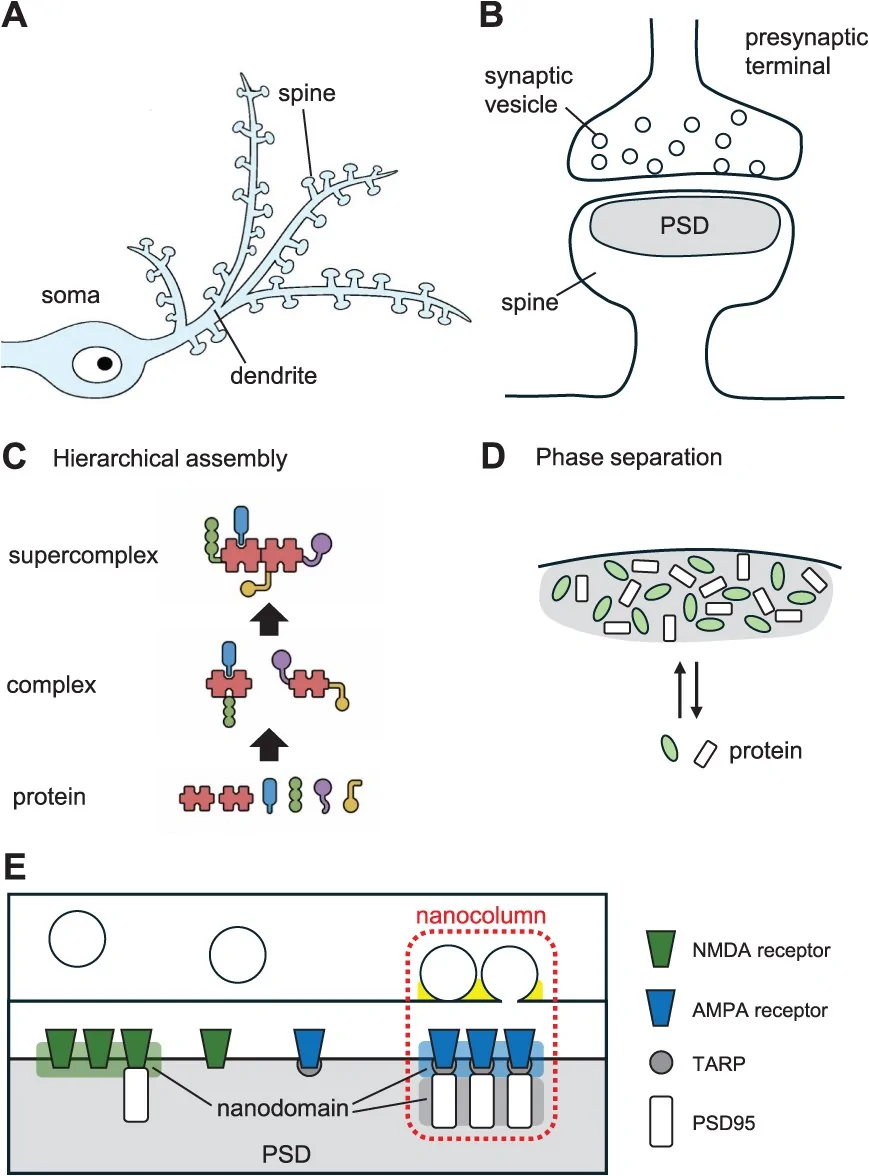
Schematic overview of an excitatory glutamatergic synapse and its postsynaptic molecular organization. (A) A neuron showing dendrites extending from the soma. Dendritic spines protrude from the dendritic shafts and form postsynaptic compartments of excitatory synapses. (B) A schematic representation of an excitatory synapse. Beneath the postsynaptic membrane, a protein-dense structure called the postsynaptic density (PSD) is located. The PSD is composed of receptors, scaffold proteins that anchor receptors, signaling enzymes, and cytoskeletal regulators. (C) Conceptual illustration of the hierarchical assembly of synaptic proteins. Individual proteins assemble into protein complexes, which can further associate to form higher-order assemblies, often referred to as supercomplexes. (D) Conceptual illustration of condensate formation through phase separation. Some synaptic scaffold proteins can form condensate-like assemblies through weak, multivalent protein–protein interactions. (E) Nanoscale molecular organization within the PSD. Synaptic proteins form subsynaptic nanodomains in which specific proteins or protein complexes are locally enriched. PSD95-containing nanodomains are often spatially associated with AMPA receptor complexes, although PSD95 can interact with both NMDA receptor complexes and AMPA receptor complexes, with the latter interaction mediated through transmembrane AMPA receptor regulatory proteins. The red dashed box illustrates a putative trans-synaptic nanocolumn, in which presynaptic release sites (yellow) are aligned with postsynaptic receptor–scaffold nanodomains.

## Postsynaptic proteome architecture

The PSD is a protein assembly located beneath the postsynaptic membrane and comprises thousands of proteins (*1**,*  *2**,*  *7–9*). Membrane-associated guanylate kinase (MAGUK) family proteins, such as postsynaptic density protein 95 (PSD95), function as central scaffolds that organize the PSD (*4*). MAGUKs bind indirectly to AMPA receptor complexes and directly to NMDA receptor complexes, anchoring them at the PSD and linking them to intracellular signaling components. MAGUKs interact with DLG-associated proteins (DLGAPs), which in turn connect to Src Homology 3 and multiple ankyrin repeat domains (Shank) proteins. Together, MAGUKs, DLGAPs, and Shank proteins create a vertical structural axis that extends from the membrane to deeper layers of the PSD. Consistent with this vertical organization, major scaffolding proteins occupy distinct laminar positions within the PSD, showing different mean distances from the postsynaptic membrane (*6**,*  *10*). Family with sequence similarity 81 member A (FAM81A), another PSD-enriched protein, interacts with multiple postsynaptic molecules, including PSD95 and the NMDA receptor subunit GluN2B (*11**,*  *12*). In addition to ionotropic glutamate receptors, there are metabotropic glutamate receptors. mGluR1 and mGluR5 couple glutamate signaling to biochemical cascades mediated by phospholipase C beta (PLCβ) (*13*). These receptors are often enriched at perisynaptic or extrasynaptic regions adjacent to the PSD, where they are linked to Shank-based postsynaptic assemblies through Homer scaffold proteins (*2**,*  *4*). It has been suggested that these PSD proteins are organized through a hierarchical assembly process (*14**,*  *15*) (Fig. 1C). In this framework, individual proteins first form defined oligomeric complexes, which subsequently associate into megadalton-scale ‘supercomplexes’ within individual synapses, as demonstrated by native biochemical approaches, including blue-native PAGE (*14**,*  *15*). In addition to this hierarchical organization, liquid–liquid phase separation (LLPS) has been proposed as another mechanism contributing to the local concentration and organization of postsynaptic proteins. In this process, multivalent protein–protein interactions drive the formation of biomolecular condensates (*11**,*  *16–20*) (Fig. 1D). Although the extent to which LLPS contributes to PSD organization *in vivo* remains under investigation, it provides a useful conceptual framework for understanding the concentration and dynamics of proteins within the PSD.

Classical electron microscopy and immunoelectron microscopy showed that the PSD is an organized protein-dense specialization and hinted that its molecular composition is not spatially uniform within a synapse (*21*). These ultrastructural observations provided an early framework for subsynaptic compartmentalization. Super-resolution imaging has revealed nanodomains of AMPA receptors, NMDA receptors, and scaffolding proteins in the PSD (*22–29*) (Fig. 1E). Moreover, postsynaptic nanodomains containing PSD95 and AMPA receptors align with presynaptic release sites to form trans-synaptic nanocolumns (*22**,*  *25*) (Fig. 1E). These structures are thought to enhance synaptic efficacy by increasing the probability that released glutamate encounters postsynaptic receptors (*24**,*  *27*). Recently, a multiplexed spatial proteomics study using high-throughput super-resolution microscopy enabled nanoscale mapping of dozens of synaptic proteins in neurons at single-protein resolution (*30*). Together, hierarchical scaffolding, phase-separated condensation, and nanoscale molecular organization provide a conceptual framework for understanding how excitatory synapses assemble and maintain their proteomic composition, and how this composition varies across brain regions, cell types, and circuits.

## Synaptic diversity uncovered by omics studies

### Investigation of synapse diversity using proteomics and synaptome mapping

Excitatory synapses are not uniform but instead exhibit remarkable diversity in their structural organization, molecular composition, physiological properties, and forms of synaptic plasticity (*31*). Dendritic spines vary widely in size, shape, and stability, and these morphological characteristics are closely linked to synaptic strength and learning-related remodeling (*3**,*  *32*). At the molecular level, the diversity of synaptic protein composition is important for understanding synaptic function and neural circuit formation. For example, glutamate receptor subunit composition shapes postsynaptic responses and synaptic plasticity (*33**,*  *34*). In addition, combinatorial interactions between pre- and postsynaptic adhesion molecules contribute to the specificity and functional properties of synaptic connections (*35*). These examples highlight the broader importance of examining how synaptic protein composition varies across individual synapses. There are multiple axes that contribute to synaptic heterogeneity across the brain, including regional variation, cell-type specificity, developmental and maturation-dependent differences, and activity- and experience-dependent remodeling. Two approaches are being used to uncover such synaptic heterogeneity: synaptic proteomics and imaging-based synaptome mapping. Proteomics uses mass spectrometry to detect and quantify hundreds to thousands of synapse-associated proteins in a largely unbiased manner (*2**,*  *36–38*) (Fig. 2A). This enables systematic comparisons across regions, ages, cell types, physiological states, and disease conditions. In practice, most datasets rely on biochemical enrichment of synaptic material, including synaptosome fractions and PSD fractions (*38*). Synaptosome fractions are biochemically isolated synaptic membrane preparations enriched in synaptosomes, which are resealed synaptic structures that retain presynaptic terminals together with apposed postsynaptic components (*38**,*  *39*). Fluorescence-activated synaptosome sorting (FASS) is a method for isolating fluorescently labeled subpopulations of synaptosomes (*40*). PSD fractions can be obtained from synaptosomes by detergent extraction (*38**,*  *41*). Compared with synaptosome fractions, PSD fractions provide stronger enrichment and a higher signal-to-noise ratio for postsynaptic scaffolding and signaling proteins (*42*). In addition to these fractionation-based methods, affinity purification and proximity labeling can enable targeted analysis of specific synaptic proteins or compartments (*36*). Affinity purification includes immunoprecipitation and peptide-mediated isolation of synaptic protein complexes (*43–46*). Proximity labeling-based proteomics uses enzymes such as biotin ligases or peroxidases fused to a bait protein or compartment-targeting motif to mediate the biotinylation of nearby proteins in living cells or tissues (*36**,*  *47**,*  *48*). The labeled proteins are subsequently enriched using streptavidin, enabling spatially resolved profiling of synaptic protein composition. This approach is becoming increasingly popular as technology continues to develop. A meta-analysis of 35 PSD proteome datasets showed that the majority of proteins captured by targeted purification of core postsynaptic components are also present in unbiased biochemically purified PSD fractions (*11*).

**Fig. 2:**
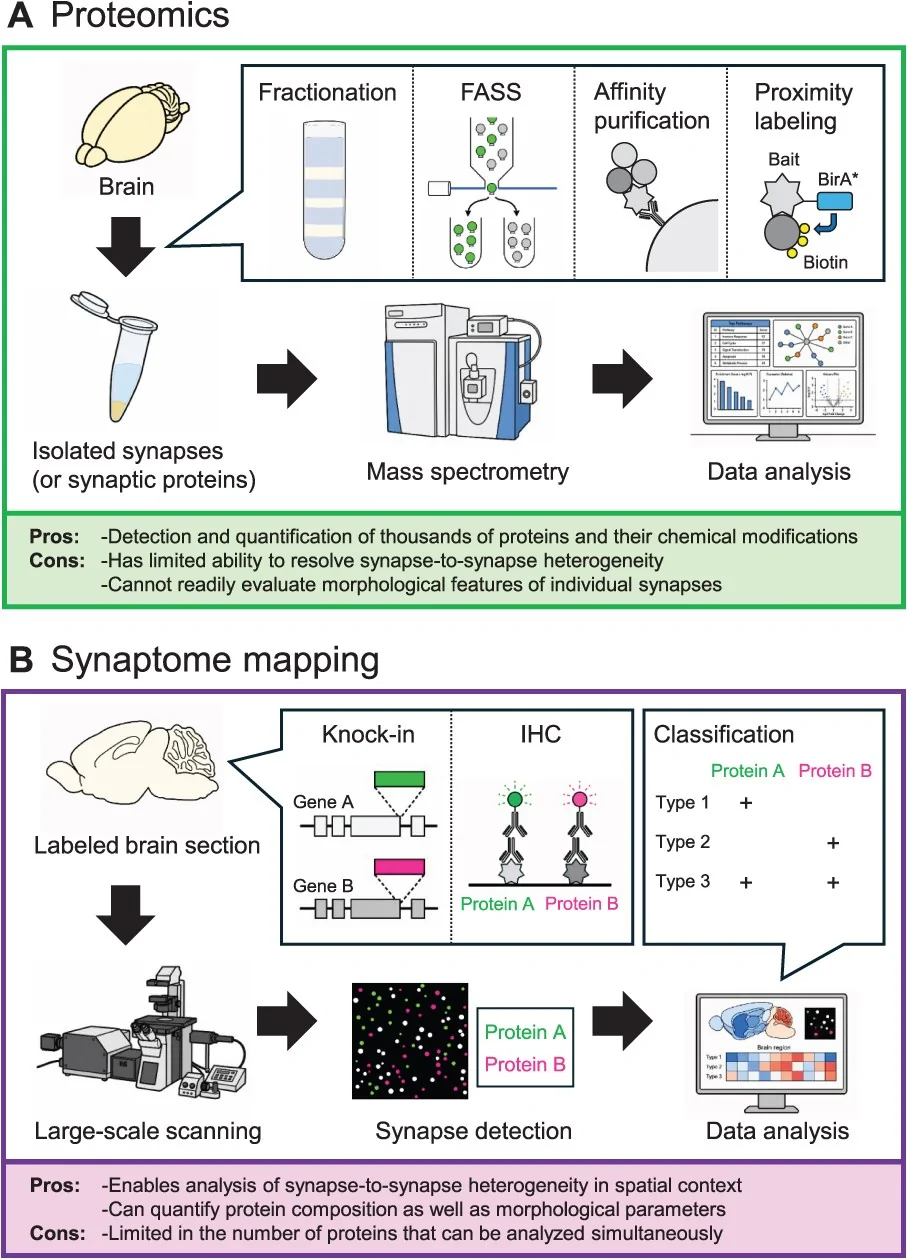
Methods to investigate diversity in synaptic protein composition. (A) Proteomics-based approach. Synaptic fractions or synaptic protein preparations derived from brain samples are subjected to mass spectrometry for protein identification and quantification. Synaptosomes and PSD fractions can be isolated by biochemical fractionation (*38**,*  *41*). Fluorescence-activated synaptosome sorting (FASS) enables the isolation of fluorescently labeled synaptosomes (*40*). Affinity purification enriches proteins physically associated with a bait molecule, while proximity labeling, using enzymes such as the mutant biotin ligase BirA*, biotinylates proteins located near a bait protein for subsequent enrichment (*36**,*  *47**,*  *48*). The resulting proteomic profiles can be further analyzed using Gene Ontology and pathway analyses to characterize detected or differentially abundant proteins. (B) Synaptome mapping. Synaptic proteins are fluorescently labeled *in situ* by genetic tagging or IHC, followed by large-scale imaging at single-synapse resolution across broad anatomical regions, up to whole-brain coverage. Computational pipelines detect synaptic puncta and extract quantitative features, including size, shape, and fluorescence intensity as a proxy for protein abundance. Synapses can be classified into distinct types based on their protein composition. In the schematic example shown here, puncta are classified as Protein A-positive, Protein B-positive, or double-positive synapses, corresponding to green, magenta, and white puncta, respectively. The resulting datasets can be used for downstream analyses, such as quantitative comparison across brain regions and experimental conditions.

The other approach, synaptome mapping, is a spatial omics strategy that resolves synapses as individual molecular entities within brain tissue sections (*49–55*) (Fig. 2B). The term ‘synaptome’ describes the set of all synapses in the brain (*49*). In synaptome mapping, synaptic proteins are visualized using genetically encoded tags or antibody-based labeling, and the brain samples are subjected to large-scale imaging to capture millions of synaptic puncta across the brain. This is followed by data processing with computational pipelines that detect and segment synapses, enabling extraction of quantitative features of synaptic size, shape, and protein abundance. The anatomical and spatial organization of diverse synapses across the brain is referred to as the ‘synaptome architecture’. Although synaptome mapping can evaluate a limited number of synaptic proteins at once, it enables the analysis of individual synapses *in situ*. More recently, synaptome mapping has been applied to single-cell analyses that comprehensively label and quantify endogenous synaptic proteins across thousands of synapses along the dendritic arbor of an individual neuron (*56*). Together, proteomics and synaptome mapping provide complementary insights into the diversity of synapses across the brain. Proteomics defines broad molecular repertoires and network-level shifts, whereas synaptome mapping reveals how these molecular identities are organized across anatomical and circuit space.

### Brain-region specificity

Synaptic diversity is prominently manifested as brain-region-specific molecular specialization. For example, quantitative proteome analysis of AMPA receptors isolated from distinct regions of the rat brain revealed region-specific differences in their GluA1–4 subunit composition (*57*).

Unbiased proteomic analyses of synaptosome fractions and PSD fractions have shown that different brain regions exhibit distinct molecular profiles across the rodent brain (*58–61*) (Fig. 3A and B). In these datasets, cortical regions show high similarity and often cluster with the hippocampus, whereas the cerebellum is consistently the most divergent (*58–61*). This tendency is also evident in the relative abundance patterns of representative postsynaptic proteins analyzed in these studies (Fig. 3C). Proteins enriched in cortical and hippocampal PSDs were associated with canonical excitatory synaptic signaling and synaptic plasticity, including glutamatergic receptor complexes and calcium signaling (*58**,*  *59*). By contrast, proteins more abundant in cerebellar PSDs were enriched for cerebellum- and Purkinje-associated organizers (*58*). In cerebellar synaptosome fractions, proteins associated with RNA metabolism, particularly mRNA processing and splicing, are more abundant than in other regions (*60*). Regional specialization is also evident within the human neocortex. Proteomic profiling of PSD fractions across 12 Brodmann areas identified area-specific molecular signatures (*62*). The observed differences include region-dependent expression of receptor subunits and MAGUK scaffolds and are organized into co-regulated protein modules, yielding distinct biochemical ‘pathway signatures’ across cortical areas. These differences support the concept that individual brain regions have a characteristic synaptic proteomic identity, reflecting how synapses are biochemically tuned to circuit function.

**Fig. 3:**
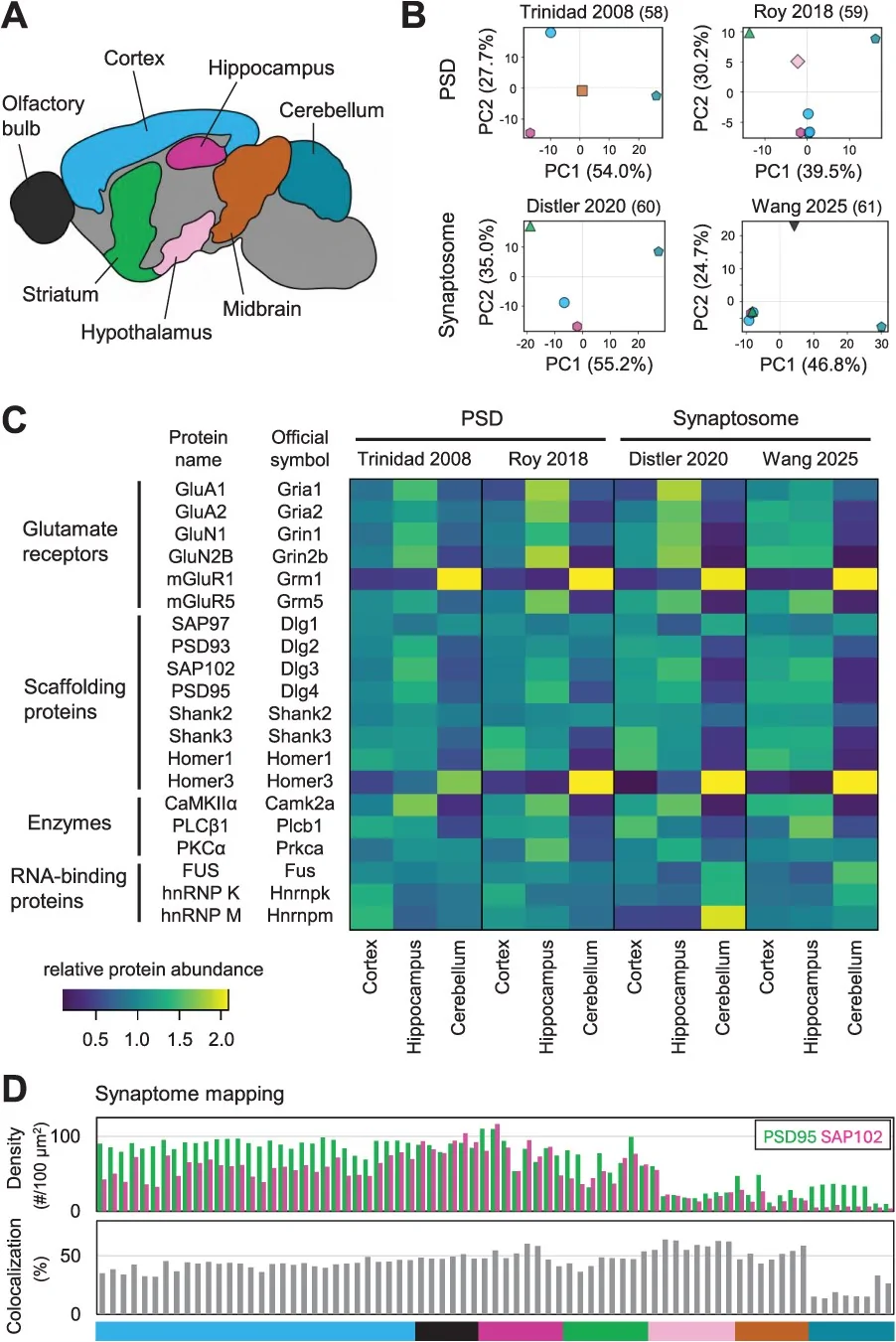
Regional diversity of synaptic protein composition in the mammalian brain. (A) Seven brain regions examined in four comparative studies of synaptic proteomes. (B) PCA-based overview of region-specific synaptic proteomic profiles. Quantitative proteomic datasets obtained from PSD fractions (*58**,*  *59*) or synaptosome fractions (*60**,*  *61*) were analyzed. For each dataset, protein abundance values were normalized so that the mean abundance across the analyzed brain regions was 1, and the normalized values were subjected to principal component analysis (PCA). Marker colors correspond to the brain regions shown in panel A. (C) Regional abundance patterns of representative synaptic proteins. Heatmaps show the normalized abundance of 20 representative proteins quantified in all four datasets. Only cortex, hippocampus, and cerebellum are shown, as these regions were included in all four studies. (D) Region-specific synaptic diversity revealed by synaptome mapping (*49*). Top, densities of PSD95-positive and SAP102-positive synapses across 75 brain regions. Bottom, percentages of synapses positive for both PSD95 and SAP102. The colored annotation bar indicates the corresponding brain regions shown in panel A.

Complementing these proteomic studies, synaptome mapping has shown regional specificity of synapses by large-scale imaging of individual synapses (Fig. 3D). Initial synaptome mapping was performed in mice expressing fluorescently tagged MAGUK proteins, PSD95 and synapse-associated protein 102 (SAP102) (*49**,*  *50*). These proteins were chosen because they are major postsynaptic scaffold proteins that mark excitatory synapses while also differing in their molecular organization. PSD95 is one of the most abundant postsynaptic scaffold proteins and interacts with multiple core postsynaptic molecules (*2*). SAP102 is a paralog of PSD95 that is also enriched in the PSD and forms supercomplexes distinct from those containing PSD95 (*14*). The synaptome mapping studies showed that each anatomical region is characterized by a distinct ‘synaptome signature’, a characteristic combination of synapse density, punctum size, and intensity (*49**,*  *50*). In addition to quantifying PSD95- and SAP102-positive synapse density, these analyses also revealed regional differences in the proportion of synapses containing both PSD95 and SAP102 (Fig. 3D). Unsupervised classification resolved discrete ‘synapse subtypes’ with stereotyped spatial distributions, and the subtype maps captured laminar and subfield structures. These include layer-specific patterns in cortex, gradients within hippocampal cornu ammonis area 1 (CA1), and compartmentalization in striatum (*49*). Synapse diversity is highest in the hippocampal formation and neocortex and lowest in the brainstem, and regional signatures cluster into forebrain, brainstem, and cerebellar blocks, consistent with a hierarchical organization across brain systems. Region-dependent differences have also been observed in the human brain by synaptome mapping of immunolabeled PSD95 (*54*). In addition to compositional differences, synaptome mapping of PSD95 protein lifetime using pulse-chase labeling of PSD95 molecules showed regional variation in excitatory synapse proteostasis (*52*). PSD95 half-life differs by roughly an order of magnitude across mouse brain subregions, with the longest lifetimes concentrated in cortical and hippocampal structures and the shortest lifetimes prominent in olfactory and many subcortical/brainstem-related regions. Regional specialization is also evident at the nanoscale. Super-resolution microscopy of PSD95 revealed diversity in synapse nanoarchitecture (*28**,*  *29*). At synapses, PSD95 is organized into conserved nanoclusters, but the number of nanoclusters per PSD differs across subregions of hippocampus (*28*) and spinal cord (*29*). In order to evaluate the diversity of synaptic nanoarchitecture at a whole-brain scale, a recent study developed a synaptome mapping pipeline in which proximity between MAGUK proteins is analyzed using Förster resonance energy transfer (FRET) (*63*). Analysis of PSD95 protein proximity revealed regional differences in protein proximity, including higher proximity in the cerebellum and superficial cortical layers.

### Cell type and circuit specificity

While synaptic composition varies across brain regions, it is also important to understand synaptic diversity defined by neuronal identity and wiring specificity. Classical and contemporary frameworks propose that synapses acquire ‘molecular identities’ through the combinatorial expression of recognition and adhesion molecules (*64**,*  *65*). Within glutamatergic transmission, excitatory synapses formed by different neuronal classes or within distinct circuits differ in their composition of receptors, scaffolds, and accessory proteins (*31*). Single-cell transcriptomic atlases have extended this view in an unbiased manner by defining neuronal cell types and uncovering cell-type-specific expression programs for synapse-related genes across cortical and hippocampal neurons, suggesting corresponding diversification at the protein level (*66**,*  *67*).

Proteomic analysis of genetically defined excitatory synapses has been accomplished by purification of glutamatergic synaptosome subpopulations using FASS (*40*). Based on this method, large-scale profiling of synapses across multiple neuronal classes showed that neuronal cell type explains a substantial fraction of variance in synaptic proteomes, in some cases exceeding the contribution of brain region (*68*). In addition to cell identity, circuit specificity also contributes to the diversity of synaptic composition. Proteomic dissection of hippocampal mossy fiber synapses revealed a specialized cell-surface proteome and identified a candidate organizer of synaptic connectivity, underscoring circuit-specific molecular diversity within glutamatergic synapses (*69*). Another study introduced a workflow based on laser-capture microdissection to obtain synaptic fractions from hippocampal layers corresponding to the trisynaptic circuit and subjected them to quantitative proteomics (*70*). This enabled direct comparison of synaptic proteomes across circuit-defined hippocampal synapse populations and revealed molecular differences, including differential expression of proteins involved in glutamate receptor regulation. A recent study combined precision microdissection, RNA sequencing, FASS, and mass spectrometry to generate an integrated transcriptomic and proteomic map of the mouse hippocampus at both tissue and synaptic levels, revealing distinct molecular signatures across hippocampal subregions and CA1 strata (*71*). More recently, a projection-specific proximity-labeling approach was used to profile presynaptic proteomes across six pathways centered on the medial prefrontal cortex (*72*). This analysis revealed marked molecular divergence among circuits, with pathway-specific enrichment of proteins involved in vesicle trafficking, cytoskeletal organization, synaptic adhesion, and intracellular signaling.

Synaptome mapping suggested that synaptic protein composition is aligned with long-range circuit organization. For example, corticothalamic and brainstem inputs to the ventral posterior thalamic nucleus were shown to form synapses with different combinations of PSD95 and SAP102 (*49*). In addition, quantitative comparisons between synaptome maps and mesoscale connectome data uncovered correlations between regional connectivity strength and the abundance of specific synapse types and subtypes (*49*). Together, these transcriptomic, proteomic, and spatial mapping studies indicate that excitatory synapses are diversified not only by anatomical region but also by neuronal identity and connectivity.

### Development and aging

Synaptic molecular architecture is not fixed but evolves across the lifespan (*1*). For example, during early postnatal development, synaptic NMDA receptors undergo a gradual developmental shift in subunit composition, with GluN2B-containing receptors becoming less predominant and GluN2A-containing receptors becoming more prominent. This change reshapes NMDA receptor signaling and is closely linked to the maturation of experience-dependent synaptic plasticity (*33*). Proteomic studies of synaptosome fractions (*73*) and PSD fractions (*74**,*  *75*) comprehensively characterized developmental changes in synaptic protein composition. Differentially expressed proteins during development include proteins related to Rho GTPase signaling (*74**,*  *75*). This is accompanied by downregulation of Rho GTPase signaling during development, which may contribute to changes in dendritic spines (*74*). Another study revealed alterations in postsynaptic protein complex composition (*43*). Proteomic analysis of immunoselected core PSD scaffolds across development revealed that postsynaptic protein complexes are extensively remodeled over time, with the embryonic cortex showing a ‘preassembly’ in which PSD95/DLGAP complexes already associate with NMDA receptor subunits but remain largely uncoupled from the Shank protein layer (*43*). As development proceeds, these scaffold interactomes gain additional modules and connectivity, indicating that postsynaptic molecular architecture is shaped not only by protein abundance but also by stage-dependent rewiring of postsynaptic protein interaction networks.

Synaptome mapping provides a brainwide, single-synapse-resolution view of developmental remodeling focusing on PSD95- and SAP102-labeled excitatory synapses (*50*). It revealed a continuous lifespan trajectory in which synapse diversity expands during early postnatal development and drives regional differentiation up to early adulthood, whereas later compositional shifts lead to progressive ‘dedifferentiation’ of brain regions in old age. As for protein turnover, the lifetime of PSD95 at synapses increases across most brain regions from juvenile stage to adulthood and further into old age (*52*). Despite these developmental and aging-associated shifts in synaptic protein composition and PSD95 turnover, the FRET-based analysis of PSD95 proximity showed a broadly conserved brainwide organization and only a modest, non-linear trajectory across the lifespan (*63*). Notably, during early maturation the proximity metric did not scale with the marked increase in PSD95 abundance, suggesting that synapses may actively buffer PSD95-PSD95 proximity. Together, these multi-omic and imaging approaches suggest that developmental processes robustly reconfigure synaptic composition, interaction networks, and protein turnover, while key nanoscale architectural constraints are comparatively preserved. This may allow synapses to adapt their signaling and plasticity repertoire across the lifespan without compromising core organizational principles of the PSD.

### Activity-, experience-, and state-dependent remodeling

Synaptic molecular architecture is dynamically tuned by physiological and behavioral states. Synaptic plasticity induces activity-dependent remodeling of the postsynaptic proteome, most prominently by changing the abundance, subunit composition, and post-translational modification state of AMPA receptors and their associated scaffold/auxiliary partners at the PSD (*34**,*  *76*). Large-scale *in vivo* imaging of endogenously tagged AMPA receptors (SEP-GluA1 knock-in) enabled single-synapse–resolution mapping of experience-evoked changes in synaptic strength, including long-term potentiation (LTP), across cortical regions (*77*). During LTP, many postsynaptic proteins including AMPA receptors undergo changes in synaptic localization. Live imaging analysis of single spines showed that postsynaptic proteins are recruited to dendritic spines with distinct kinetics during LTP induced by glutamate uncaging (*78*). Quantitative PSD proteomics after chemical LTP (cLTP) revealed selective changes in synaptic protein abundance, including a prominent increase in RNA-binding proteins (*79*). Subsequent studies extended this ‘abundance-change’ view by using proteomics to map remodeling of post-translational modifications (*80–82*). In mouse hippocampal CA1 slices, LTP induction altered phosphorylation at 570 sites across 222 PSD proteins, with LTP-regulated phosphoproteins enriched among highly connected nodes in the core PSD scaffold interactome, consistent with rapid reorganization of scaffold-centered signaling networks during potentiation (*80*). Palmitoylome profiling identified 121 hippocampal proteins with learning-dependent changes in palmitoylation, and a subset of these proteins showed similar activity-dependent palmitoylation after cLTP (*81*). This study also showed that palmitoylation of plasticity-related gene-1 (PRG-1) supports activity-induced AMPA receptor insertion into the postsynaptic membrane. Another study reported that cLTP induces activity-dependent post-translational regulation of zinc-finger DHHC-domain containing (ZDHHC) palmitoyl transferases, modulating their stability, protein interactions, and enzymatic activity (*82*). These results suggest that regulated palmitoylation contributes to synaptic potentiation. Beyond experimentally induced synaptic plasticity, endogenous brain states also remodel synaptic molecular architecture. In particular, sleep–wake cycles are associated with state-dependent changes in synaptic protein composition and phosphorylation. Proteome analysis of PSD fractions demonstrated that sleep promotes Homer1a-dependent homeostatic scaling-down of excitatory synapses (*83*). Phosphoproteomic analysis of synaptosomes collected at six time points revealed dynamic changes in synaptic protein phosphorylation across the sleep–wake cycle (*84*).

Brainwide synaptome mapping of PSD95- and SAP102-labeled excitatory synapses showed that the distributions of synapse types and subtypes are largely stable across the normal sleep–wake cycle, but just 6 hours of sleep deprivation rapidly remodels synaptome architecture specifically in the cortex and hippocampal CA1 (*53*). Sleep deprivation reduced the size of PSD95-positive synapses and decreased excitatory synapse subtype diversity without changing overall synapse density, producing a subtype redistribution biased toward synapses with long protein lifetimes (with a corresponding loss of synapses with short protein lifetimes) that was predicted to alter circuit responses to activity patterns such as theta/gamma and sharp-wave ripple-like inputs (*53*). Using the same knock-in mouse line, a recent brainwide synaptome mapping study showed that environmental enrichment and developmental monocular deprivation each drive large-scale, experience-specific reprogramming of excitatory synapse type and subtype populations across brain regions, without a corresponding global increase in total synapse density (*85*). These distinct, spatially distributed signatures were aligned with mesoscale connectome organization, supporting a model in which experience is encoded as a brainwide trace via selective remodeling of synapse varieties.

### Disease

Neurodegenerative, neuropsychiatric, and neurodevelopmental disorders are associated with diverse synaptic abnormalities, including aberrant synaptic pruning, altered morphology of dendritic spines, and impaired synaptic transmission or plasticity (*86**,*  *87*). This is consistent with the observation that many disease-associated proteins localize at synapses. SynaptopathyDB, an integrative database of synaptic proteins, reports 516 disease-associated postsynaptic proteins, 206 disease-associated presynaptic proteins, and 514 disease-associated synaptosome proteins, highlighting the extensive burden of pathogenic variants in genes encoding synaptic proteins (*7*). Proteomic analyses of synaptic fractions derived from the brains of patients and animal models have revealed alterations in synaptic protein composition in neurodegenerative, neuropsychiatric, and neurodevelopmental disorders (Fig. 4). A recent study performed proteomic analysis of synapse-enriched fractions from three cortical regions of patients with Alzheimer’s disease (ad) and identified both shared and region-specific alterations in the synaptic proteome (*88*). Region-specific changes included reduced abundance of proteins related to inhibitory synapses in the prefrontal cortex, downregulation of mitochondria-related pathways in the visual and prefrontal cortices, and broader alterations in pathways associated with synaptic organization and function in the middle temporal gyrus. In patients with ad and psychosis, the PSD proteome in the dorsolateral prefrontal cortex (DLPFC) showed reduced abundance of 240 proteins, including proteins related to Rho GTPase signaling (*89*). By contrast, proteomic analysis of hippocampal synaptosomes from 12-month-old ad model mice showed increased abundance of 30 proteins, including proteins related to microglia-mediated synapse pruning (*90*). Proteomic analyses of DLPFC synaptosomes from patients with schizophrenia (SCZ) and bipolar disorder (BD) showed convergent synaptic pathology, characterized by overlapping decreases in PSD components and increases in vesicle-trafficking proteins (*91*). The same study also analyzed A-kinase anchoring protein 11 (*Akap11*)-deficient mice. *Akap11* encodes A-kinase anchoring protein 11 (AKAP11), and its deficiency can be regarded as a model relevant to both SCZ and BD. Although the overall proteomic changes in the cortex showed poor correlation with those observed in human patients, pathway-level similarities were detected, including increased abundance of vesicle-trafficking proteins (*91*). In contrast, analyses of PSD fractions from the anterior cingulate cortex of patients with SCZ or BD showed reduced abundance of proteins associated with calcium signaling in SCZ and increased abundance of mitochondrial proteins in BD (*92**,*  *93*). The differences between SCZ and BD observed in these studies may reflect disease-specific protein signatures in the anterior cingulate cortex or disease-dependent differences in protein localization to the PSD. Proteomic analyses of DLPFC synaptosomes from patients with autism spectrum disorder (ASD) showed an overall decrease in PSD components, particularly during childhood, whereas analysis of cerebellar synaptosomes revealed changes including decreased abundance of vesicle-trafficking proteins (*94**,*  *95*). Among animal models of ASD, one study compared hippocampal PSD samples from five different ASD model mouse lines. Comparison of differentially expressed proteins across individual lines suggested commonly affected pathways in ASD mouse models, including proteins related to Rho GTPase signaling (*96*). Another PSD proteome study using *Shank3* mutant mice, a model of ASD, revealed brain region-specific changes in PSD protein composition (*97*). Whereas the hippocampal PSD showed altered abundance of proteins mainly associated with cytoskeletal organization, the striatal PSD showed a predominant decrease in proteins involved in glutamatergic synaptic transmission, including core components of the PSD.

**Fig. 4:**
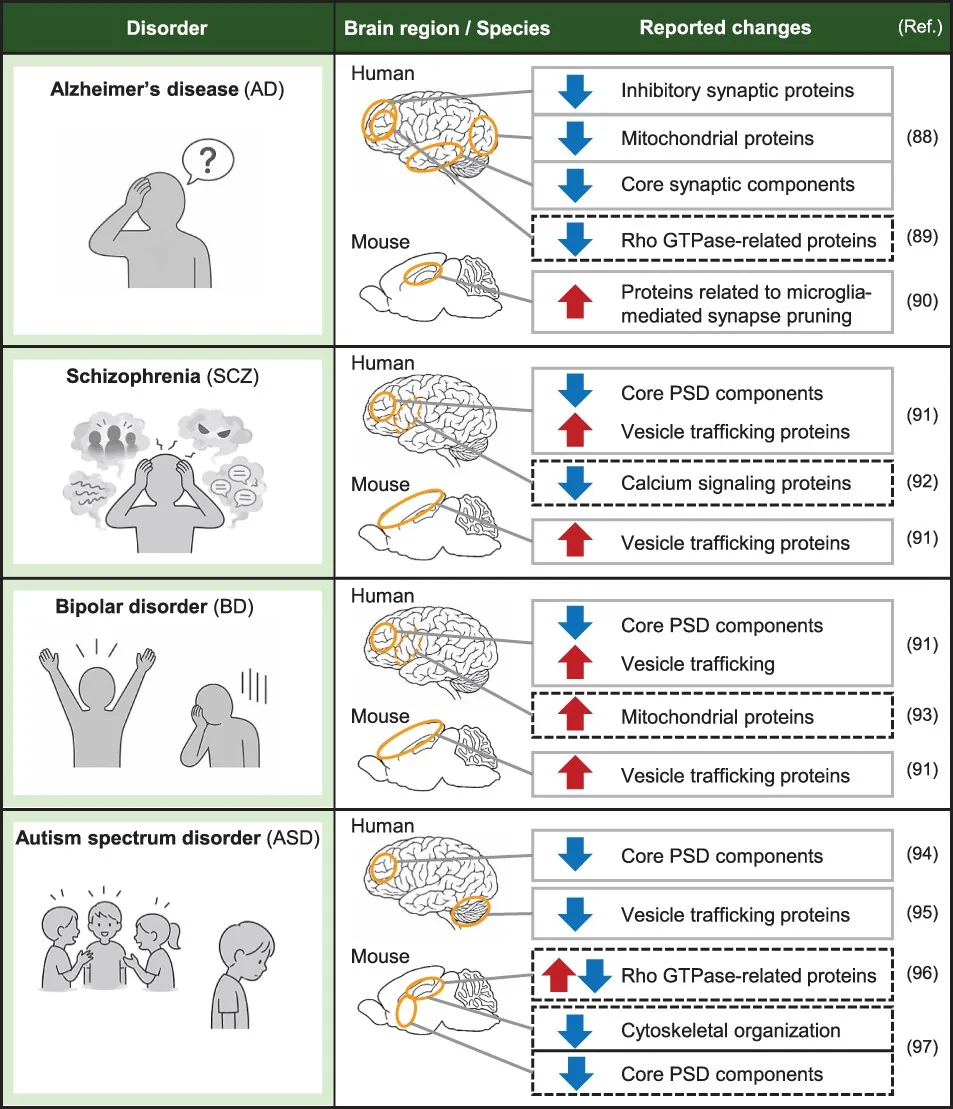
Reported changes in synaptic protein composition in neurodegenerative, neuropsychiatric, and neurodevelopmental disorders. This figure summarizes studies reporting alterations in synaptic protein composition in the brains of patients or mouse models of ad, SCZ, BD, and ASD. For each disorder, the brain regions and species analyzed are indicated in the middle column, and representative reported changes in synaptic protein abundance are shown in the right column. Downward and upward arrows indicate decreased and increased abundance, respectively. Studies using synaptosome fractions are indicated by boxes with light gray solid lines, whereas studies using postsynaptic density (PSD) fractions are indicated by boxes with dark gray dashed lines.

Synaptome mapping provides a complementary viewpoint by detecting disease-associated changes as shifts in the abundance or spatial distribution of synapse subtypes within defined circuits. In a schizophrenia-relevant mouse model lacking *Dlg2*, which encodes the postsynaptic protein PSD93, single-synapse synaptome mapping revealed widespread synaptome reprogramming (*49*). Regionally patterned changes in PSD95 puncta parameters reorganized PSD95 synaptome network topology, increasing regional similarity, clustering, modularity, and small-worldness. These changes were also associated with impaired pattern-specific synaptome responses in hippocampal CA1 (*49*). Loss of PSD93 also robustly increases PSD95 lifetime at synapses, prolonging the PSD95 half-life across all 110 brain regions in a gene-dosage-dependent manner (*52*). Analysis of synaptic nanoarchitecture shows that loss of PSD93 causes a robust brainwide increase in the proximity between PSD95 molecules, consistent with a reorganization toward more tightly packed PSD95 assemblies within individual excitatory synapses (*63*). In the murine neurodevelopmental disease model caused by *Pax6* haploinsufficiency, brainwide synaptome mapping shows that total excitatory synapse number is unchanged, but the molecular composition of excitatory synapses is altered such that the normal postnatal expansion of synapse subtype diversity and maturation of synaptome architecture are delayed across brain regions (*51*). These abnormalities emerge transiently in two developmental ‘phenotype waves’ (around P7–P21 and again around P35–P42) and then spontaneously reverse within approximately 2–3 weeks, restoring synapse diversity and synaptome architecture toward the normal developmental trajectory. Recently, single-cell synaptome mapping using dual labeling of surface and intracellular GluA2 in neurons that lack synaptic Ras GTPase-activating protein (SynGAP) was reported (*56*). SynGAP functions as a GTPase-activating protein (GAP) for small GTPases. Its encoding gene *Syngap1* is associated with neurodevelopmental disorders, including ASD. The results of single-cell synaptome mapping revealed a widespread reduction of the intracellular AMPA receptor pool with a concomitant increase in the surface-to-intracellular GluA2 ratio across the majority of synapses, suggesting a potential mechanism underlying AMPA receptor-related plasticity phenotypes in SynGAP deficiency (*56*). Together, proteomics and synaptome mapping provide complementary views of synaptic disease biology. Proteomics pinpoints the molecular modules and pathways that are perturbed, whereas synaptome mapping reveals how these perturbations manifest *in vivo* as circuit- and region-specific shifts in synapse subtype composition and spatial organization.

## Future perspectives

Synapses are often introduced as stereotyped transmission units, yet the evidence summarized in this review shows that they are better understood as molecularly diversified structures whose composition, organization, and dynamics are tuned across anatomical space and biological time. Across brain regions, synapses adopt characteristic proteomic signatures and structural features, while at finer scales they reflect neuronal identity and long-range wiring. Moreover, synaptic composition is not static. It is reshaped during postnatal development and aging, reconfigured by activity and experience, and perturbed in disease. Together, these observations indicate that the spatial and molecular organization of synapse populations represents a key dimension of brain organization, with important implications for understanding how neuronal function is specialized, adapted, and disrupted across regions, life stages, and disease states.

Proteomics and imaging-based synaptome mapping are both powerful tools for uncovering the molecular composition and organization of synapses and their diversity. Recent methodological advances are providing access to synaptic data that were not obtained previously. In particular, proteome profiling of FASS-isolated synaptosome subpopulations, proximity-labeling-based proteomics, and newly developed imaging-based synaptome mapping are expected to help delineate how synapses are remodeled across diverse physiological and pathological conditions. Among these approaches, synaptome mapping is still at an early stage, and most current applications focus on a small set of postsynaptic markers, such as PSD95 and SAP102. A major reason is the technical difficulty of labeling endogenous proteins comprehensively in intact brain tissue. At present, labeling is typically achieved via knock-in mouse lines that enable genetically encoded tagging of target proteins. Looking ahead, complementary strategies, such as adeno-associated virus (AAV)-mediated genome editing to introduce tags *in vivo*, should also be explored to expand flexibility and throughput. Expanding the set of target proteins to include other postsynaptic proteins, presynaptic proteins, and markers of inhibitory synapses is expected to deepen our understanding of synaptic protein composition and its diversity across brain regions and states. In addition to knock-in labeling, classical immunohistochemistry (IHC) can also be used to label endogenous proteins for synaptome mapping (*54**,*  *55*). In practice, IHC would be the first-choice approach for studies of human brain tissue. For large-scale quantitative analyses, however, it is important to pay careful attention to false positives and false negatives. Synaptic proteins can be challenging to detect by conventional immunohistochemical methods (*98**,*  *99*). When IHC is adopted for synaptome mapping, it is critical to optimize the experimental conditions using appropriate controls.

Comprehensive analysis of synapses using proteomics and synaptome mapping should not only refine our understanding of synaptic organization, but also help uncover molecular mechanisms that link synaptic change to neural function and to the pathogenesis of neurological, neuropsychiatric, and neurodevelopmental disorders. It will also be important to investigate how psychotropic medications reshape synapses at the systems level. For many drugs currently used in psychiatry, the primary pharmacological targets are known, yet the mechanisms by which these initial actions translate into sustained therapeutic benefit remain incompletely understood. State-of-the-art proteomics and synaptome mapping offer a powerful route to dissect these multi-step pathways *in situ* and to connect drug-induced molecular remodeling to synaptic architecture across circuits. Taken together, these emerging approaches provide an important framework for linking synaptic molecular remodeling to circuit function and to the pathogenesis of neurological, neuropsychiatric, and neurodevelopmental disorders.

It should be noted that both approaches have pros and cons (Fig. 2). A major strength of proteomics is its breadth: it can quantify thousands of proteins and their chemical modifications, providing an unbiased view of coordinated changes and pathway-level remodeling across conditions. However, because proteomic measurements typically rely on pooled material, they largely report population averages across vast numbers of synapses, making it difficult in principle to resolve the defining features of individual synapses. Moreover, achieving high spatial resolution remains challenging. By contrast, synaptome mapping captures synapse-to-synapse heterogeneity directly and links molecular signatures to spatial context across regions and circuits. In addition, it can report not only protein composition but also nanoscale organization and morphological features of synapses, such as synapse size and shape, which are generally not directly accessible using bulk proteomic approaches. However, current implementations are restricted in molecular breadth: the number of proteins that can be measured simultaneously is typically limited to one or a few, constraining how fully synaptic proteomes and their combinatorial diversity can be reconstructed. In the future, it will be important to develop techniques that mitigate the weaknesses of each modality and to perform integrative analyses that connect the complementary information they provide. For example, multiplex protein labeling could enable synaptome mapping of many more targets (*30**,*  *100**,*  *101*). Although the application of multiplex labeling to brain tissue sections remains challenging, this approach could extend *in situ* synapse classification from a handful of markers toward richer molecular repertoires.

In summary, synaptic protein organization provides a molecular substrate for the brain’s functional specialization, adaptability, and vulnerability. By jointly leveraging proteomics and synaptome mapping, and by connecting these approaches to nanoscale imaging, circuit analysis, and perturbation experiments, the field is converging on a more integrated understanding of how synapses are built, maintained, diversified, and remodeled across the lifespan and in disease.

## References

[1] Kaizuka, T. and Takumi, T. (2024) Alteration of synaptic protein composition during developmental synapse maturation. Eur. J. Neurosci. 59, 2894–2914 10.1111/ejn.16304 38571321

[2] Kaizuka, T. and Takumi, T. (2018) Postsynaptic density proteins and their involvement in neurodevelopmental disorders. *J. Biochem.* 163, 447–455 10.1093/jb/mvy022 29415158

[3] Kasai, H., Ziv, N.E., Okazaki, H., Yagishita, S., and Toyoizumi, T. (2021l) Spine dynamics in the brain, mental disorders and artificial neural networks. Nat. Rev. Neurosci. 22, 407–422 10.1038/s41583-021-00467-3 34050339

[4] Zhu, J., Shang, Y., and Zhang, M. (2016) Mechanistic basis of MAGUK-organized complexes in synaptic development and signalling. Nat. Rev. Neurosci. 17, 209–223 10.1038/nrn.2016.18 26988743

[5] Dolphin, A.C. and Lee, A. (2020) Presynaptic calcium channels: specialized control of synaptic neurotransmitter release. Nat. Rev. Neurosci. 21, 213–229 10.1038/s41583-020-0278-2 PMC7873717 32161339

[6] Dosemeci, A., Weinberg, R.J., Reese, T.S., and Tao-Cheng, J.H. (2016) The postsynaptic density: there is more than meets the eye. Front Synaptic Neurosci. 8, 23 10.3389/fnsyn.2016.00023 PMC4990544 27594834

[7] Sorokina, O., Dominic, D., Bayés, À., Armstrong, J.D., and Grant, S.G.N. (2025) SynaptopathyDB integrates synaptic proteomes, genetic and phenotypic data to advance research on nervous system disorders. Sci. Rep. 15, 42986 10.1038/s41598-025-26969-z PMC12673098 41330977

[8] Bayés, À., Van De Lagemaat, L.N., Collins, M.O., Croning, M.D.R., Whittle, I.R., Choudhary, J.S., and Grant, S.G. (2011) Characterization of the proteome, diseases and evolution of the human postsynaptic density. Nat. Neurosci. 14, 19–21 10.1038/nn.2719 PMC3040565 21170055

[9] Bayés, À., Collins, M.O., Croning, M.D.R., Van De Lagemaat, L.N., Choudhary, J.S., and Grant, S.G.N. (2012) Comparative study of human and mouse postsynaptic proteomes finds high compositional conservation and abundance differences for key synaptic proteins. PLoS One 7, e46683 10.1371/journal.pone.0046683 PMC3465276 23071613

[10] Liu, Y.T., Tao, C.L., Lau, P.M., Zhou, Z.H., and Bi, G.Q. (2019) Postsynaptic protein organization revealed by electron microscopy. Curr. Opin. Struct. Biol. 54, 152–160 10.1016/j.sbi.2019.02.012 PMC6753054 30904821

[11] Kaizuka, T., Hirouchi, T., Saneyoshi, T., Shirafuji, T., Collins, M.O., Grant, S.G.N., Hayashi, Y., and Takumi, T. (2024) FAM81A is a postsynaptic protein that regulates the condensation of postsynaptic proteins via liquid–liquid phase separation. PLoS Biol. 22, e3002006 10.1371/journal.pbio.3002006 PMC10919877 38452102

[12] Dosemeci, A., Loo, H.K., Toy, D., Winters, C.A., Reese, T.S., and Tao-Cheng, J.H. (2019) FAM81A protein, a novel component of the postsynaptic density in adult brain. Neurosci. Lett. 699, 122–126 10.1016/j.neulet.2019.02.003 PMC6443461 30735723

[13] Mao, L.M., Bodepudi, A., Chu, X.P., and Wang, J.Q. (2022) Group I metabotropic glutamate receptors and interacting partners: an update. Int. J. Mol. Sci. 23, 840 10.3390/ijms23020840 PMC8778124 35055030

[14] Frank, R.A.W., Komiyama, N.H., Ryan, T.J., Zhu, F., O’Dell, T.J., and Grant, S.G.N. (2016) NMDA receptors are selectively partitioned into complexes and supercomplexes during synapse maturation. Nat. Commun. 7, 11264 10.1038/ncomms11264 PMC5227094 27117477

[15] Frank, R.A. and Grant, S.G. (2017) Supramolecular organization of NMDA receptors and the postsynaptic density. Curr. Opin. Neurobiol. 45, 139–147 10.1016/j.conb.2017.05.019 PMC5557338 28577431

[16] Zeng, M., Shang, Y., Araki, Y., Guo, T., Huganir, R.L., and Zhang, M. (2016) Phase transition in postsynaptic densities underlies formation of synaptic complexes and synaptic plasticity. Cell. 166, 1163–1175.e12 10.1016/j.cell.2016.07.008 PMC5564291 27565345

[17] Zeng, M., Chen, X., Guan, D., Xu, J., Wu, H., Tong, P., and Zhang, M. (2018) Reconstituted postsynaptic density as a molecular platform for understanding synapse formation and plasticity. Cell. 174, 1172–1187.e16 10.1016/j.cell.2018.06.047 30078712

[18] Zeng, M., Díaz-Alonso, J., Ye, F., Chen, X., Xu, J., Ji, Z., Nicoll, R.A., and Zhang, M. (2019) Phase separation-mediated TARP/MAGUK complex condensation and AMPA receptor synaptic transmission. Neuron. 104, 529–543.e6 10.1016/j.neuron.2019.08.001 PMC6842113 31492534

[19] Cai, Q., Zeng, M., Wu, X., Wu, H., Zhan, Y., Tian, R., and Zhang, M. (2021) CaMKIIα-driven, phosphatase-checked postsynaptic plasticity via phase separation. Cell Res. 31, 37–51 10.1038/s41422-020-00439-9 PMC7852677 33235361

[20] Hosokawa, T., Liu, P.W., Cai, Q., Ferreira, J.S., Levet, F., Butler, C., Sibarita, J.B., Choquet, D., Groc, L., Hosy, E., Zhang, M., and Hayashi, Y. (2021) CaMKII activation persistently segregates postsynaptic proteins via liquid phase separation. Nat. Neurosci. 24, 777–785 10.1038/s41593-021-00843-3 33927400

[21] Harris, K.M. and Weinberg, R.J. (2012) Ultrastructure of synapses in the mammalian brain. Cold Spring Harb. Perspect. Biol. 4, a005587 10.1101/cshperspect.a005587 PMC3331701 22357909

[22] Biederer, T., Kaeser, P.S., and Blanpied, T.A. (2017) Transcellular nanoalignment of synaptic function. Neuron. 96, 680–696 10.1016/j.neuron.2017.10.006 PMC5777221 29096080

[23] MacGillavry, H.D., Song, Y., Raghavachari, S., and Blanpied, T.A. (2013) Nanoscale scaffolding domains within the postsynaptic density concentrate synaptic AMPA receptors. Neuron. 78, 615–622 10.1016/j.neuron.2013.03.009 PMC3668352 23719161

[24] Ramsey, A.M., Tang, A.H., LeGates, T.A., Gou, X.Z., Carbone, B.E., Thompson, S.M., Biederer, T., and Blanpied, T.A. (2021) Subsynaptic positioning of AMPARs by LRRTM2 controls synaptic strength. Sci. Adv. 7, eabf3126 10.1126/sciadv.abf3126 PMC8378824 34417170

[25] Tang, A.H., Chen, H., Li, T.P., Metzbower, S.R., MacGillavry, H.D., and Blanpied, T.A. (2016) A trans-synaptic nanocolumn aligns neurotransmitter release to receptors. Nature. 536, 210–214 10.1038/nature19058 PMC5002394 27462810

[26] Kellermayer, B., Ferreira, J.S., Dupuis, J., Levet, F., Grillo-Bosch, D., Bard, L., Linarès-Loyez, J., Bouchet, D., Choquet, D., Rusakov, D.A., Bon, P., Sibarita, J.B., Cognet, L., Sainlos, M., Carvalho, A.L., and Groc, L. (2018) Differential nanoscale topography and functional role of GluN2-NMDA receptor subtypes at glutamatergic synapses. Neuron. 100, 106–119.e7 10.1016/j.neuron.2018.09.012 30269991

[27] Haas, K.T., Compans, B., Letellier, M., Bartol, T.M., Grillo-Bosch, D., Sejnowski, T.J., Sainlos, M., Choquet, D., Thoumine, O., and Hosy, E. (2018) Pre-post synaptic alignment through neuroligin-1 tunes synaptic transmission efficiency. eLife. 7, e31755 10.7554/eLife.31755 PMC6070337 30044218

[28] Broadhead, M.J., Horrocks, M.H., Zhu, F., Muresan, L., Benavides-Piccione, R., DeFelipe, J., Fricker, D., Kopanitsa, M.V., Duncan, R.R., Klenerman, D., Komiyama, N.H., Lee, S.F., and Grant, S.G.N. (2016) PSD95 nanoclusters are postsynaptic building blocks in hippocampus circuits. Sci. Rep. 6, 24626 10.1038/srep24626 PMC4842999 27109929

[29] Broadhead, M.J., Bonthron, C., Arcinas, L., Bez, S., Zhu, F., Goff, F., Nylk, J., Dholakia, K., Gunn-Moore, F., Grant, S.G.N., and Miles, G.B. (2020) Nanostructural diversity of synapses in the mammalian spinal cord. Sci. Rep. 10, 8189 10.1038/s41598-020-64874-9 PMC7235094 32424125

[30] Unterauer, E.M., Shetab Boushehri, S., Jevdokimenko, K., Masullo, L.A., Ganji, M., Sograte-Idrissi, S., Kowalewski, R., Strauss, S., Reinhardt, S.C.M., Perovic, A., Marr, C., Opazo, F., Fornasiero, E.F., and Jungmann, R. (2024) Spatial proteomics in neurons at single-protein resolution. Cell. 187, 1785–1800.e16 10.1016/j.cell.2024.02.045 38552614

[31] O’Rourke, N.A., Weiler, N.C., Micheva, K.D., and Smith, S.J. (2012) Deep molecular diversity of mammalian synapses: why it matters and how to measure it. Nat. Rev. Neurosci. 13, 365–379 10.1038/nrn3170 PMC3670986 22573027

[32] Berry, K.P. and Nedivi, E. (2017) Spine dynamics: are they all the same? Neuron. 96, 43–55 10.1016/j.neuron.2017.08.008 PMC5661952 28957675

[33] Paoletti, P., Bellone, C., and Zhou, Q. (2013) NMDA receptor subunit diversity: impact on receptor properties, synaptic plasticity and disease. Nat. Rev. Neurosci. 14, 383–400 10.1038/nrn3504 23686171

[34] Diering, G.H. and Huganir, R.L. (2018) The AMPA receptor code of synaptic plasticity. Neuron. 100, 314–329 10.1016/j.neuron.2018.10.018 PMC6214363 30359599

[35] Südhof, T.C. (2021) The cell biology of synapse formation. J. Cell Biol. 220, e202103052 10.1083/jcb.202103052 PMC8186004 34086051

[36] Van Gelder, C.A.G.H. and Altelaar, M. (2021) Neuroproteomics of the synapse: subcellular quantification of protein networks and signaling dynamics. Mol. Cell. Proteomics 20, 100087 10.1016/j.mcpro.2021.100087 PMC8167277 33933679

[37] Van Oostrum, M. and Schuman, E.M. (2025) Understanding the molecular diversity of synapses. Nat. Rev. Neurosci. 26, 65–81 10.1038/s41583-024-00888-w 39638892

[38] Dieterich, D.C. and Kreutz, M.R. (2016) Proteomics of the synapse – a quantitative approach to neuronal plasticity. Mol. Cell. Proteomics 15, 368–381 10.1074/mcp.R115.051482 PMC4739661 26307175

[39] Evans, G.J.O. (2015) The synaptosome as a model system for studying synaptic physiology. Cold Spring Harb Protoc 2015, pdb.top074450 10.1101/pdb.top074450 25934942

[40] Biesemann, C., Grønborg, M., Luquet, E., Wichert, S.P., Bernard, V., Bungers, S.R., Cooper, B., Varoqueaux, F., Li, L., Byrne, J.A., Urlaub, H., Jahn, O., Brose, N., and Herzog, E. (2014) Proteomic screening of glutamatergic mouse brain synaptosomes isolated by fluorescence activated sorting. EMBO J. 33, 157–170 10.1002/embj.201386120 PMC3989609 24413018

[41] Carlin, R.K., Grab, D.J., Cohen, R.S., and Siekevitz, P. (1980) Isolation and characterization of postsynaptic densities from various brain regions: enrichment of different types of postsynaptic densities. J. Cell Biol. 86, 831–845 10.1083/jcb.86.3.831 PMC2110694 7410481

[42] Pandya, N.J., Koopmans, F., Slotman, J.A., Paliukhovich, I., Houtsmuller, A.B., Smit, A.B., and Li, K.W. (2017) Correlation profiling of brain sub-cellular proteomes reveals co-assembly of synaptic proteins and subcellular distribution. Sci. Rep. 7, 12107 10.1038/s41598-017-11690-3 PMC5608747 28935861

[43] Li, J., Zhang, W., Yang, H., Howrigan, D.P., Wilkinson, B., Souaiaia, T., Evgrafov, O.V., Genovese, G., Clementel, V.A., Tudor, J.C., Abel, T., Knowles, J.A., Neale, B.M., Wang, K., Sun, F., and Coba, M.P. (2017) Spatiotemporal profile of postsynaptic interactomes integrates components of complex brain disorders. Nat. Neurosci. 20, 1150–1161 10.1038/nn.4594 PMC5645082 28671696

[44] Bayés, À., Collins, M.O., Galtrey, C.M., Simonnet, C., Roy, M., Croning, M.D., Gou, G., van de Lagemaat, L.N., Milward, D., Whittle, I.R., Smith, C., Choudhary, J.S., and Grant, S.G.N. (2014) Human post-mortem synapse proteome integrity screening for proteomic studies of postsynaptic complexes. Mol Brain 7, 88 10.1186/s13041-014-0088-4 PMC4271336 25429717

[45] Fernández, E., Collins, M.O., Uren, R.T., Kopanitsa, M.V., Komiyama, N.H., Croning, M.D.R., Zografos, L., Armstrong, J.D., Choudhary, J.S., and Grant, S.G. (2009) Targeted tandem affinity purification of PSD-95 recovers core postsynaptic complexes and schizophrenia susceptibility proteins. Mol. Syst. Biol. 5, 269 10.1038/msb.2009.27 PMC2694677 19455133

[46] Husi, H., Ward, M.A., Choudhary, J.S., Blackstock, W.P., and Grant, S.G.N. (2000) Proteomic analysis of NMDA receptor–adhesion protein signaling complexes. Nat. Neurosci. 3, 661–669 10.1038/76615 10862698

[47] Ito, Y., Nagamoto, S., and Takano, T. (2024) Synaptic proteomics decode novel molecular landscape in the brain. Front. Mol. Neurosci. 17, 1361956 10.3389/fnmol.2024.1361956 PMC11079194 38726307

[48] Matsubayashi, J. and Takano, T. (2025) Proximity labeling uncovers the synaptic proteome under physiological and pathological conditions. Front. Cell. Neurosci. 19, 1638627 10.3389/fncel.2025.1638627 PMC12325354 40771565

[49] Zhu, F., Cizeron, M., Qiu, Z., Benavides-Piccione, R., Kopanitsa, M.V., Skene, N.G., Koniaris, B., DeFelipe, J., Fransén, E., Komiyama, N.H., and Grant, S.G.N. (2018) Architecture of the mouse brain synaptome. Neuron. 99, 781–799.e10 10.1016/j.neuron.2018.07.007 PMC6117470 30078578

[50] Cizeron, M., Qiu, Z., Koniaris, B., Gokhale, R., Komiyama, N.H., Fransén, E., and Grant, S.G.N. (2020) A brainwide atlas of synapses across the mouse life span. Science. 369, 270–275 10.1126/science.aba3163 PMC7115813 32527927

[51] Tomas-Roca, L., Qiu, Z., Fransén, E., Gokhale, R., Bulovaite, E., Price, D.J., Komiyama, N.H., and Grant, S.G.N. (2022) Developmental disruption and restoration of brain synaptome architecture in the murine Pax6 neurodevelopmental disease model. Nat. Commun. 13, 6836 10.1038/s41467-022-34131-w PMC9652404 36369219

[52] Bulovaite, E., Qiu, Z., Kratschke, M., Zgraj, A., Fricker, D.G., Tuck, E.J., Gokhale, R., Koniaris, B., Jami, S.A., Merino-Serrais, P., Husi, E., Mendive-Tapia, L., Vendrell, M., O’Dell, T.J., DeFelipe, J., Komiyama, N.H., Holtmaat, A., Fransén, E., and Grant, S.G.N. (2022) A brain atlas of synapse protein lifetime across the mouse lifespan. Neuron. 110, 4057–4073.e8 10.1016/j.neuron.2022.09.009 PMC9789179 36202095

[53] Koukaroudi, D., Qiu, Z., Fransén, E., Gokhale, R., Bulovaite, E., Komiyama, N.H., Seibt, J., and Grant, S.G.N. (2024) Sleep maintains excitatory synapse diversity in the cortex and hippocampus. Curr. Biol. 34, 3836–3843.e5 10.1016/j.cub.2024.07.032 PMC11359089 39096907

[54] Curran, O.E., Qiu, Z., Smith, C., and Grant, S.G.N. (2021) A single-synapse resolution survey of PSD95-positive synapses in twenty human brain regions. Eur. J. Neurosci. 54, 6864–6881 10.1111/ejn.14846 PMC7615673 32492218

[55] Wong, T., Qiu, Z., Notman, B., Tavares, A., Smith, C., and Grant, S.G.N. (2025) SV2A is expressed in synapse subpopulations in mouse and human brain: implications for PET radiotracer studies. Wellcome Open Res. 10, 415 10.12688/wellcomeopenres.24668.1 PMC12603525 41229902

[56] Uchigashima, M., Iguchi, R., Fujii, K., Shiku, K., Kumar, P., Liu, X., Isogai, M., Hoshino, C., Abe, M., Nozumi, M., Okamura, Y., Igarashi, M., Sakimura, K., Bise, R., Lavis, L.D., and Mikuni, T. (2025) Single-cell synaptome mapping of endogenous protein subpopulations in mammalian brain. Nat. Commun. 16, 9705 10.1038/s41467-025-65813-w PMC12606364 41219189

[57] Schwenk, J., Baehrens, D., Haupt, A., Bildl, W., Boudkkazi, S., Roeper, J., Fakler, B., and Schulte, U. (2014) Regional diversity and developmental dynamics of the AMPA-receptor proteome in the mammalian brain. Neuron. 84, 41–54 10.1016/j.neuron.2014.08.044 25242221

[58] Trinidad, J.C., Thalhammer, A., Specht, C.G., Lynn, A.J., Baker, P.R., Schoepfer, R., and Burlingame, A.L. (2008) Quantitative analysis of synaptic phosphorylation and protein expression. Mol. Cell. Proteomics 7, 684–696 10.1074/mcp.M700170-MCP200 18056256

[59] Roy, M., Sorokina, O., McLean, C., Tapia-González, S., DeFelipe, J., Armstrong, J.D., and Grant, S.G.N. (2018) Regional diversity in the postsynaptic proteome of the mouse brain. Proteomes. 6, 31 10.3390/proteomes6030031 PMC6161190 30071621

[60] Distler, U., Schumann, S., Kesseler, H.G., Pielot, R., Smalla, K.H., Sielaff, M., Schmeisser, M.J., and Tenzer, S. (2020) Proteomic analysis of brain region and sex-specific synaptic protein expression in the adult mouse brain. Cells. 9, 313 10.3390/cells9020313 PMC7072627 32012899

[61] Wang, Y., Li, Y., Tian, X., Wu, Y., Li, Y., Zhao, X., Cai, Y., Zhang, P., Zhang, W., and Kong, D. (2025) A resource for exploring regional diversity in the male rat brain synaptic proteome. Sci. Data 12, 1494 10.1038/s41597-025-05873-1 PMC12391371 40866373

[62] Roy, M., Sorokina, O., Skene, N., Simonnet, C., Mazzo, F., Zwart, R., Sher, E., Smith, C., Armstrong, J.D., and Grant, S.G.N. (2018) Proteomic analysis of postsynaptic proteins in regions of the human neocortex. Nat. Neurosci. 21, 130–138 10.1038/s41593-017-0025-9 29203896

[63] Kaizuka, T., Qiu, Z., Bulovaite, E., Morris, K., Zhao, T., Adams, C., Varga, G., Dominic, D., Komiyama, N.H., Horrocks, M.H., and Grant, S.G.N. (2026) A brainwide atlas of synaptic nanoarchitecture across the mouse lifespan. bioRxiv. 10.64898/2026.02.18.706606

[64] Sanes, J.R. and Yamagata, M. (2009) Many paths to synaptic specificity. Annu. Rev. Cell Dev. Biol. 25, 161–195 10.1146/annurev.cellbio.24.110707.175402 19575668

[65] Sanes, J.R. and Zipursky, S.L. (2020) Synaptic specificity, recognition molecules, and assembly of neural circuits. Cell. 181, 536–556 10.1016/j.cell.2020.04.008 32359437

[66] Tasic, B., Yao, Z., Graybuck, L.T., Smith, K.A., Nguyen, T.N., Bertagnolli, D., Goldy, J., Garren, E., Economo, M.N., Viswanathan, S., Penn, O., Bakken, T., Menon, V., Miller, J., Fong, O., Hirokawa, K.E., Lathia, K., Rimorin, C., Tieu, M., Larsen, R., Casper, T., Barkan, E., Kroll, M., Parry, S., Shapovalova, N.V., Hirschstein, D., Pendergraft, J., Sullivan, H.A., Kim, T.K., Szafer, A., Dee, N., Groblewski, P., Wickersham, I., Cetin, A., Harris, J.A., Levi, B.P., Sunkin, S.M., Madisen, L., Daigle, T.L., Looger, L., Bernard, A., Phillips, J., Lein, E., Hawrylycz, M., Svoboda, K., Jones, A.R., Koch, C., and Zeng, H. (2018) Shared and distinct transcriptomic cell types across neocortical areas. Nature. 563, 72–78 10.1038/s41586-018-0654-5 PMC6456269 30382198

[67] Zeisel, A., Muñoz-Manchado, A.B., Codeluppi, S., Lönnerberg, P., La Manno, G., Juréus, A., Marques, S., Munguba, H., He, L., Betsholtz, C., Rolny, C., Castelo-Branco, G., Hjerling-Leffler, J., and Linnarsson, S. (2015) Cell types in the mouse cortex and hippocampus revealed by single-cell RNA-seq. Science. 347, 1138–1142 10.1126/science.aaa1934 25700174

[68] Van Oostrum, M., Blok, T.M., Giandomenico, S.L., Tom Dieck, S., Tushev, G., Fürst, N., Langer, J.D., and Schuman, E.M. (2023) The proteomic landscape of synaptic diversity across brain regions and cell types. Cell. 186, 5411–5427.e23 10.1016/j.cell.2023.09.028 PMC10686415 37918396

[69] Apóstolo, N., Smukowski, S.N., Vanderlinden, J., Condomitti, G., Rybakin, V., Ten Bos, J., Trobiani, L., Portegies, S., Vennekens, K.M., Gounko, N.V., Comoletti, D., Wierda, K.D., Savas, J.N., and de Wit, J. (2020) Synapse type-specific proteomic dissection identifies IgSF8 as a hippocampal CA3 microcircuit organizer. Nat. Commun. 11, 5171 10.1038/s41467-020-18956-x PMC7560607 33057002

[70] Reig-Viader, R., Del Castillo-Berges, D., Burgas-Pau, A., Arco-Alonso, D., Zerpa-Rios, O., Ramos-Vicente, D., Picañol, J., Castellanos, A., Soto, D., Roher, N., Sindreu, C., and Bayés, À. (2025) Synaptic proteome diversity is shaped by the levels of glutamate receptors and their regulatory proteins. Nat. Commun. 16, 10487 10.1038/s41467-025-65490-9 PMC12647736 41290593

[71] Kaulich, E., Waselenchuk, Q., Fürst, N., Desch, K., Mosbacher, J., Ciirdaeva, E., Juengling, M., Ray, R., Nassim-Assir, B., Tushev, G., Langer, J.D., and Schuman, E.M. (2025) An integrated transcriptomic and proteomic map of the mouse hippocampus at synaptic resolution. Nat. Commun. 16, 7942 10.1038/s41467-025-63119-5 PMC12381143 40858553

[72] Ito, Y., Nagamoto, S., Uchiyama, S., Onishi, K., Matsubayashi, J., Fukuda, A., Funakoshi, M., Hattori, S., Takeuchi, K., Kudo, M., Masuda, T., Hiraoka, Y., Kohro, Y., Shimogori, T., Tsuda, M., Kosako, H., and Takano, T. (2026) Spatial proteomics reveals prefrontal circuit diversity in socioemotional behaviour. bioRxiv. 10.64898/2026.02.08.704617

[73] Gonzalez-Lozano, M.A., Klemmer, P., Gebuis, T., Hassan, C., Van Nierop, P., Van Kesteren, R.E., Smit, A.B., and Li, K.W. (2016) Dynamics of the mouse brain cortical synaptic proteome during postnatal brain development. Sci. Rep. 6, 35456 10.1038/srep35456 PMC5066275 27748445

[74] Kaizuka, T., Suzuki, T., Kishi, N., Tamada, K., Kilimann, M.W., Ueyama, T., Watanabe, M., Shimogori, T., Okano, H., Dohmae, N., and Takumi, T. (2024) Remodeling of the postsynaptic proteome in male mice and marmosets during synapse development. Nat. Commun. 15, 2496 10.1038/s41467-024-46529-9 PMC10979008 38548776

[75] Wang, L., Pang, K., Zhou, L., Cebrián-Silla, A., González-Granero, S., Wang, S., Bi, Q., White, M.L., Ho, B., Li, J., Li, T., Perez, Y., Huang, E.J., Winkler, E.A., Paredes, M.F., Kovner, R., Sestan, N., Pollen, A.A., Liu, P., Li, J., Piao, X., García-Verdugo, J.M., Alvarez-Buylla, A., Liu, Z., and Kriegstein, A.R. (2023) A cross-species proteomic map reveals neoteny of human synapse development. Nature. 622, 112–119 10.1038/s41586-023-06542-2 PMC10576238 37704727

[76] Chater, T.E. and Goda, Y. (2014) The role of AMPA receptors in postsynaptic mechanisms of synaptic plasticity. Front. Cell. Neurosci. 8, 401 10.3389/fncel.2014.00401 PMC4245900 25505875

[77] Graves, A.R., Roth, R.H., Tan, H.L., Zhu, Q., Bygrave, A.M., Lopez-Ortega, E., Hong, I., Spiegel, A.C., Johnson, R.C., Vogelstein, J.T., Tward, D.J., Miller, M.I., and Huganir, R.L. (2021) Visualizing synaptic plasticity in vivo by large-scale imaging of endogenous AMPA receptors. eLife. 10, e66809 10.7554/eLife.66809 PMC8616579 34658338

[78] Bosch, M., Castro, J., Saneyoshi, T., Matsuno, H., Sur, M., and Hayashi, Y. (2014) Structural and molecular remodeling of dendritic spine substructures during long-term potentiation. Neuron. 82, 444–459 10.1016/j.neuron.2014.03.021 PMC4281348 24742465

[79] Zhang, G., Neubert, T.A., and Jordan, B.A. (2012) RNA binding proteins accumulate at the postsynaptic density with synaptic activity. J. Neurosci. 32, 599–609 10.1523/JNEUROSCI.2463-11.2012 PMC3561928 22238095

[80] Li, J., Wilkinson, B., Clementel, V.A., Hou, J., O’Dell, T.J., and Coba, M.P. (2016) Long-term potentiation modulates synaptic phosphorylation networks and reshapes the structure of the postsynaptic interactome. Sci. Signal. 9, rs8 10.1126/scisignal.aaf6716 27507650

[81] Nasseri, G.G., Matin, N., Wild, A.R., Tosefsky, K., Flibotte, S., Stacey, R.G., Hollman, R.B., Foster, L.J., and Bamji, S.X. (2022) Synaptic activity–dependent changes in the hippocampal palmitoylome. Sci. Signal. 15, eadd2519 10.1126/scisignal.add2519 36473050

[82] Abazari, D., Wild, A.R., Qiu, T., Dickinson, B.C., and Bamji, S.X. (2023) Activity-dependent post-translational regulation of palmitoylating and depalmitoylating enzymes in the hippocampus. J. Cell Sci. 136, jcs260629 10.1242/jcs.260629 PMC10113885 37039765

[83] Diering, G.H., Nirujogi, R.S., Roth, R.H., Worley, P.F., Pandey, A., and Huganir, R.L. (2017) Homer1a drives homeostatic scaling-down of excitatory synapses during sleep. Science. 355, 511–515 10.1126/science.aai8355 PMC5382711 28154077

[84] Brüning, F., Noya, S.B., Bange, T., Koutsouli, S., Rudolph, J.D., Tyagarajan, S.K., Cox, J., Mann, M., Brown, S.A., and Robles, M.S. (2019) Sleep-wake cycles drive daily dynamics of synaptic phosphorylation. Science. 366, eaav3617 10.1126/science.aav3617 31601740

[85] Woods, H., Günar, Ü., Gillard, S., Notman, B., Yuan, K., Dominic, D., Robson, E., Varga, G., Komiyama, N.H., Qiu, Z., Sengpiel, F., and Grant, S.G.N. (2025) Experiences are encoded by brainwide reprogramming of synaptome architecture. bioRxiv. 10.64898/2025.12.12.693919

[86] Dejanovic, B., Sheng, M., and Hanson, J.E. (2024) Targeting synapse function and loss for treatment of neurodegenerative diseases. Nat. Rev. Drug Discov. 23, 23–42 10.1038/s41573-023-00823-1 38012296

[87] Faust, T.E., Gunner, G., and Schafer, D.P. (2021) Mechanisms governing activity-dependent synaptic pruning in the developing mammalian CNS. Nat. Rev. Neurosci. 22, 657–673 10.1038/s41583-021-00507-y PMC8541743 34545240

[88] Griffiths, J., Schneegans, E., Whitwell, H., Qiu, Z., Notman, B., Cheung, D., Willumsen, N., Matthews, P.M., Grant, S.G.N., and Jackson, J.S. (2025) A synaptic-astrocytic proteomic signature associated with synaptopathy in Alzheimer’s disease. bioRxiv. 10.1101/2025.01.23.634408

[89] Krivinko, J.M., DeChellis-Marks, M.R., Zeng, L., Fan, P., Lopez, O.L., Ding, Y., Wang, L., Kofler, J., MacDonald, M.L., and Sweet, R.A. (2023) Targeting the post-synaptic proteome has therapeutic potential for psychosis in Alzheimer disease. Commun Biol. 6, 598 10.1038/s42003-023-04961-5 PMC10238472 37268664

[90] Van Der Spek, S.J.F., Gonzalez-Lozano, M.A., Koopmans, F., Miedema, S.S.M., Paliukhovich, I., Smit, A.B., and Li, K.W. (2021) Age-dependent hippocampal proteomics in the APP/PS1 Alzheimer mouse model: a comparative analysis with classical SWATH/DIA and directDIA approaches. Cells. 10, 1588 10.3390/cells10071588 PMC8304546 34202490

[91] Aryal, S., Bonanno, K., Song, B., Mani, D.R., Keshishian, H., Carr, S.A., Sheng, M., and Dejanovic, B. (2023) Deep proteomics identifies shared molecular pathway alterations in synapses of patients with schizophrenia and bipolar disorder and mouse model. Cell Rep. 42, 112497 10.1016/j.celrep.2023.112497 37171958

[92] Föcking, M., Lopez, L.M., English, J.A., Dicker, P., Wolff, A., Brindley, E., Wynne, K., Cagney, G., and Cotter, D.R. (2015) Proteomic and genomic evidence implicates the postsynaptic density in schizophrenia. Mol. Psychiatry 20, 424–432 10.1038/mp.2014.63 25048004

[93] Föcking, M., Dicker, P., Lopez, L.M., Hryniewiecka, M., Wynne, K., English, J.A., Cagney, G., and Cotter, D.R. (2016) Proteomic analysis of the postsynaptic density implicates synaptic function and energy pathways in bipolar disorder. Transl. Psychiatry 6, e959 10.1038/tp.2016.224 PMC5290351 27898073

[94] Fatemi, S.H., Eschenlauer, A., Aman, J., Folsom, T.D., and Chekouo, T. (2024) Quantitative proteomics of dorsolateral prefrontal cortex reveals an early pattern of synaptic dysmaturation in children with idiopathic autism. Cereb. Cortex 34, 161–171 10.1093/cercor/bhae044 PMC11484494 38696595

[95] Fatemi, S.H., Folsom, T.D., Eschenlauer, A., and Chekouo, T. (2025) Impaired aggrephagy, interrupted vesicular trafficking, and cellular stress, lead to protein aggregation, and synaptic dysfunction in cerebellum of children and adults with idiopathic autism. Cerebellum 24, 140 10.1007/s12311-025-01880-5 PMC12334520 40779003

[96] Carbonell, A.U., Freire-Cobo, C., Deyneko, I.V., Dobariya, S., Erdjument-Bromage, H., Clipperton-Allen, A.E., Page, D.T., Neubert, T.A., and Jordan, B.A. (2023) Comparing synaptic proteomes across five mouse models for autism reveals converging molecular similarities including deficits in oxidative phosphorylation and rho GTPase signaling. Front. Aging Neurosci. 15, 1152562 10.3389/fnagi.2023.1152562 PMC10225639 37255534

[97] Reim, D., Distler, U., Halbedl, S., Verpelli, C., Sala, C., Bockmann, J., Tenzer, S., Boeckers, T.M., and Schmeisser, M.J. (2017) Proteomic analysis of post-synaptic density fractions from Shank3 mutant mice reveals brain region specific changes relevant to autism spectrum disorder. Front. Mol. Neurosci. 10, 26 10.3389/fnmol.2017.00026 PMC5306440 28261056

[98] Fukaya, M. and Watanabe, M. (2000) Improved immunohistochemical detection of postsynaptically located PSD-95/SAP90 protein family by protease section pretreatment: a study in the adult mouse brain. J. Comp. Neurol. 426, 572–586 11027400

[99] Konno, K., Yamasaki, M., Miyazaki, T., and Watanabe, M. (2023) Glyoxal fixation: an approach to solve immunohistochemical problem in neuroscience research. Sci. Adv. 9, eadf7084 10.1126/sciadv.adf7084 PMC10348680 37450597

[100] Guo, S.M., Veneziano, R., Gordonov, S., Li, L., Danielson, E., Perez De Arce, K., Park, D., Kulesa, A.B., Wamhoff, E.C., Blainey, P.C., Boyden, E.S., Cottrell, J.R., and Bathe, M. (2019) Multiplexed and high-throughput neuronal fluorescence imaging with diffusible probes. Nat. Commun. 10, 4377 10.1038/s41467-019-12372-6 PMC6763432 31558769

[101] Kang, J., Schroeder, M.E., Lee, Y., Kapoor, C., Yu, E., Tarr, T.B., Titterton, K., Zeng, M., Park, D., Niederst, E., Wei, D., Feng, G., and Boyden, E.S. (2024) Multiplexed expansion revealing for imaging multiprotein nanostructures in healthy and diseased brain. Nat. Commun. 15, 9722 10.1038/s41467-024-53729-w PMC11550395 39521775

